# Assessment of the Quality and Readability of Information Provided by ChatGPT in Relation to the Use of Platelet-Rich Plasma Therapy for Osteoarthritis

**DOI:** 10.3390/jpm14050495

**Published:** 2024-05-08

**Authors:** Stephen Fahy, Marcel Niemann, Peter Böhm, Tobias Winkler, Stephan Oehme

**Affiliations:** 1 Center for Musculoskeletal Surgery, Charité—Universitätsmedizin Berlin, Corporate Member of Freie Universität Berlin and Humboldt-Universität zu Berlin, 10117 Berlin, Germany; stephen.fahy@charite.de (S.F.); marcel.niemann@charite.de (M.N.); tobias.winkler@charite.de (T.W.); 2 Deutsche Rheuma-Liga e.V., 53111 Bonn, Germany; peboehm@gmx.de; 3 Berlin Institute of Health Center for Regenerative Therapies, Berlin Institute of Health at Charité—Universitätsmedizin Berlin, 13353 Berlin, Germany; 4 Berlin Institute of Health at Charité—Universitätsmedizin Berlin, Julius Wolff Institute, 13353 Berlin, Germany

**Keywords:** osteoarthritis (OA), regenerative medicine, platelet-rich plasma (PRP), readability, digital health literacy, patient education materials (PEMs), artificial intelligence (AI)

## Abstract

**Objective**: This study aimed to evaluate the quality and readability of information generated by ChatGPT versions 3.5 and 4 concerning platelet-rich plasma (PRP) therapy in the management of knee osteoarthritis (OA), exploring whether large language models (LLMs) could play a significant role in patient education. **Design:** A total of 23 common patient queries regarding the role of PRP therapy in knee OA management were presented to ChatGPT versions 3.5 and 4. The quality of the responses was assessed using the DISCERN criteria, and readability was evaluated using six established assessment tools. **Results:** Both ChatGPT versions 3.5 and 4 produced moderate quality information. The quality of information provided by ChatGPT version 4 was significantly better than version 3.5, with mean DISCERN scores of 48.74 and 44.59, respectively. Both models scored highly with respect to response relevance and had a consistent emphasis on the importance of shared decision making. However, both versions produced content significantly above the recommended 8th grade reading level for patient education materials (PEMs), with mean reading grade levels (RGLs) of 17.18 for ChatGPT version 3.5 and 16.36 for ChatGPT version 4, indicating a potential barrier to their utility in patient education. **Conclusions:** While ChatGPT versions 3.5 and 4 both demonstrated the capability to generate information of moderate quality regarding the role of PRP therapy for knee OA, the readability of the content remains a significant barrier to widespread usage, exceeding the recommended reading levels for PEMs. Although ChatGPT version 4 showed improvements in quality and source citation, future iterations must focus on producing more accessible content to serve as a viable resource in patient education. Collaboration between healthcare providers, patient organizations, and AI developers is crucial to ensure the generation of high quality, peer reviewed, and easily understandable information that supports informed healthcare decisions.

## 1. Introduction

Osteoarthritis (OA) is a leading cause of disability globally, significantly impacting both the function and quality of life of those affected. It is estimated that approximately 15% of the global population are affected by OA [1]. The estimated lifetime risk for knee OA is 40% in males and 47% in females, with variables such as increasing age, obesity, and female gender recognized as risk factors [2]. A host of treatment strategies exist for the management of OA, ranging from non-invasive approaches, such as weight management, pharmacological interventions, and physiotherapy, to joint injections with hyaluronic acid, corticosteroids, and platelet-rich plasma (PRP), and, finally, to surgical interventions in the form of osteotomies, and unicompartmental or total knee arthroplasty.

In recent times, the field of regenerative medicine has shown potential for the treatment of a host of orthopedic conditions. Specifically, PRP has been investigated for its role in facilitating fracture healing, cartilage repair, osteoarthritis management, as well as a potential adjunctive therapy option following soft tissue surgeries, such as anterior cruciate ligament (ACL) reconstruction, rotator cuff repair, and Achilles tendon repair [3]. Unfortunately, huge variability is seen in the literature with respect to study quality, the methodology, as well as the magnitude of the therapeutic response observed. This has led to organizations, such as the American Academy of Orthopaedic Surgeons (AAOS), being reluctant to advocate the widespread use of PRP, emphasizing the need for improvements in study design, as well as standardization of the methodology used in studies [3]. Nevertheless, PRP therapy has seen huge growth in popularity in the last few years, with a global market net worth of USD 275 million in 2020, and a predicted market growth of 11% per annum [4]. This growth in popularity is often fueled by factually inaccurate claims made on the internet regarding the purported indications for, and benefits of, PRP therapy. Research has found that the most frequently accessed websites containing information about PRP therapy are often of poor quality and written at a level that far exceeds the literacy of the general public [5]. The presence of both factually inaccurate and highly complex information creates a significant problem for healthcare professionals, as it leads to patients developing both a poor understanding of the indications for PRP therapy, as well as unrealistic expectations regarding its therapeutic effects.

Despite the presence of potentially misleading information available on the internet, it is viewed as a valuable resource for information gathering among patients [6]. In modern healthcare, decision-making has shifted from the traditional paternalistic model to a shared decision-making model between physician and patient. For patients to actively participate in their healthcare decisions, they must have the ability to access, understand, and use medical information online, a skill set known as digital health literacy. It is vital that this information is scientifically accurate, or it runs the risk of negatively impacting the decision-making process. The cornerstone of digital health literacy is the capability of patients to read and comprehend online resources effectively. The average American reads at an 8th grade level (13–14 years old) and, as such, expert groups advocate that patient education materials (PEMs) are written at the 6th grade level (11 to 12 years old) to maximize readability. Despite these recommendations, numerous studies have identified that internet-based PEMs often exceed the advised reading grade levels (RGLs), significantly hindering their usefulness in aiding patients’ decision-making processes online [7,8,9,10,11,12].

Artificial intelligence (AI) tools, like ChatGPT, have become increasingly popular in recent times, and may represent a beneficial and underutilized tool for patient education. Tools equipped with natural language processing capabilities, such as ChatGPT, can be used by patients to provide immediate access to individualized healthcare information narrowing the existing knowledge gap between patients and healthcare providers. To date, very few studies have evaluated the quality and readability of data produced by AI resources in the field of orthopedics [13,14,15]. As such, our research aimed to assess the quality and readability of information produced by ChatGPT regarding PRP therapy in the management of knee OA. Furthermore, we sought to determine whether there was a discernible disparity in the quality and readability of content produced by ChatGPT versions 3.5 and 4. We hypothesized that owing to the maturation of the ChatGPT model, significant improvements would exist in the quality of the information provided.

## 2. Materials and Methods

On 6 February 2024, the natural language processing tool, ChatGPT (OpenAI Global LLC, San Francisco, CA, USA), was posed 23 common patient queries relating to the role of PRP therapy in the management of knee OA. These questions were derived from prior research, which examined the quality of internet-based patient resources relating to PRP therapy [5,16,17]. The internet resources cited in these papers addressed the most common patient-related queries concerning PRP and OA, which we selected for analysis in our study. Questions relating to the use of PRP for the management of other conditions were not included in the research. The questions were written in the first person, simulating a patient query, and deliberately written at, or below, the average 8th grade American literacy level (Appendix A). Prior to the commencement of the study, the cookies were cleared on the computer to prevent potential bias in the answers given based on previous browsing histories. Both ChatGPT version 3.5 and ChatGPT version 4 were given the same questions and the responses were saved in Microsoft Word documents. The quality of the information provided by both ChatGPT version 3.5 and version 4 was assessed by three named authors (SF, SO, MN), all of whom are registrars in orthopedics, working in the field of regenerative medicine and knee surgery.

The DISCERN criteria were used to assess the quality of the information provided by both ChatGPT models. The DISCERN criteria consist of 16 questions, each rated from 1–5. The DISCERN criteria are commonly used to assess the information quality in medicine [18]. The first eight questions assess the reliability of the content, while the next seven questions assess the information provided regarding treatment choices, the final question is an overall rating of the perceived quality. The maximum score is 80, with scores of 70 and above deemed “excellent”, and scores of 50 and above deemed “good” [13].

To assess the readability of the responses, the Readability Studio Professional Edition Program (Oleander Software Ltd., version 2019) was used [19]. This software evaluates readability with 6 assessment tools (Appendix B): the Simple Measure of Gobbledygook (SMOG) score, the Flesch Reading Ease Score (FRES), the Fry Score, the Gunning Fog Score, the Flesch–Kincaid Reading Grade Level (FKGL), and the Raygor Estimate. The reading grade levels (RGLs) were reported as the standard United States (US) grade level, frequently used in the field of readability studies [7,8]. The RGL is an estimation of the level of education and literacy required to read, understand, and retain the information contained within an article [20,21,22]. The FRES index expresses readability as an index score ranging from 0 to 100, with higher scores indicating easier readability.

Descriptive statistics were produced using GraphPad Prism 10 for macOS (version 10.1.1), while interrater reliability was assessed with IBM SPSS Statistics (version 29.0.0.0), applying the intraclass correlation coefficient in a two-way mixed model. The Wilcoxon matched pairs signed rank test was used to assess for statistically significant between-group differences in the mean total DISCERN criteria score, the mean score per DISCERN criteria category, and the readability.

This study required no ethical approval as no patient-level data was used.

## 3. Results

A typical example of the answers provided by both ChatGPT version 3.5 and ChatGPT version 4 is provided below:

*Does PRP work best if I have mild, moderate, or severe arthritis?*

### 3.1. ChatGPT Version 3.5

The effectiveness of platelet-rich plasma (PRP) therapy for knee arthritis can vary depending on the severity of the arthritis, but PRP may offer benefits across a spectrum of disease severity. However, the optimal response to PRP treatment may differ depending on the individual characteristics of the patient and the specific goals of the treatment.

Here is a general overview of how PRP therapy may work across different stages of arthritis severity:

- Mild arthritis: In patients with mild arthritis, PRP therapy may be particularly effective in addressing early symptoms, such as occasional pain, stiffness, and mild functional limitations. By promoting tissue repair, reducing inflammation, and modulating the healing process, PRP injections may help alleviate symptoms and delay disease progression in individuals with mild joint damage.
- Moderate arthritis: Patients with moderate arthritis may also benefit from PRP therapy, although the degree of symptom relief and functional improvement may vary depending on the extent of the joint damage and other factors. PRP injections may help mitigate pain, improve joint function, and enhance the overall quality of life in individuals with moderate arthritis, potentially delaying the need for more invasive treatments, such as surgery.
- Severe arthritis: In cases of severe arthritis, with significant joint damage and structural deformity, PRP therapy may have more limited effectiveness in terms of symptom management and disease modification. While PRP injections may still provide some degree of pain relief and functional improvement, the benefits may be less pronounced compared to milder cases of arthritis. In severe arthritis, PRP therapy may be used as part of a multimodal treatment approach to address symptoms and support overall joint health.

It’s important to recognize that the response to PRP therapy can vary among individuals, and factors such as age, overall health, activity level, and individual biological response to treatment can influence treatment outcomes. Additionally, the goals of treatment may differ based on the patient’s preferences, lifestyle, and functional needs.

Overall, PRP therapy may offer benefits for patients across different stages of arthritis severity, but treatment plans should be personalized based on individual patient characteristics and treatment goals. It’s essential to discuss your condition and treatment options with a qualified healthcare provider, who can provide personalized recommendations and guidance tailored to your specific needs.

### 3.2. ChatGPT Version 4

Platelet-rich plasma (PRP) therapy has been studied for its effectiveness in treating osteoarthritis (OA) of various severities, with the consensus suggesting that it may be most effective in the early stages of arthritis, which typically corresponds to mild-to-moderate OA. Heres how PRP therapy aligns with different stages of arthritis:

#### 3.2.1. Mild-to-Moderate Arthritis

- Most beneficial: PRP therapy is generally considered most beneficial for patients with mild-to-moderate osteoarthritis. At these stages, the degenerative changes in the joint are less severe, and the potential for tissue repair and symptom relief are higher. PRP therapy can potentially reduce inflammation and stimulate healing processes in the joint, improving function, and reducing pain.
- Evidence: Several studies have indicated that patients with mild-to-moderate OA tend to respond better to PRP treatments, showing improvements in pain, stiffness, and joint function. The rationale is that the natural regenerative processes can be more effectively supported when the overall structure of the joint is relatively intact.

#### 3.2.2. Severe Arthritis

- Limited effectiveness: In cases of severe arthritis, where there is significant cartilage loss and joint degeneration, PRP therapy may be less effective. At this advanced stage, the structural damage to the joint may be too extensive for PRP therapy to effectively reverse, or significantly improve, the situation through its regenerative capabilities.
- Considerations: For severe OA, more invasive treatments such as joint replacement surgery might be more appropriate and recommended by healthcare providers. PRP therapy could still be considered for symptom management in certain cases, but expectations regarding the outcomes should be realistic.

#### 3.2.3. Individual Variability

- Patient-specific factors: The effectiveness of PRP therapy can also depend on individual patient factors, including age, overall health, activity level, and the specific joint affected. These factors can influence how well someone might respond to PRP treatment, regardless of the arthritis severity.

#### 3.2.4. Consultation with a Healthcare Provider

- Before deciding on PRP therapy, it’s crucial for patients to consult with healthcare providers who can assess the severity of arthritis, discuss potential benefits and limitations of PRP therapy, and consider other medical conditions or treatments that might affect outcomes. An informed decision can be made by weighing the severity of OA against the potential benefits and limitations of PRP therapy, in the context of the individual’s overall treatment plan.

In summary, PRP injections have been most effective in treating mild-to-moderate knee osteoarthritis. Research has shown that PRP therapy can reduce pain, improve joint function, and potentially slow down the progression of osteoarthritis. The benefits of PRP injections have been observed to last up to two years for some patients, though individual results can vary. The effectiveness of PRP therapy in severe arthritis is less clear, and more research is needed to establish its benefits for high-grade osteoarthritis. For detailed information, you may refer to the American Academy of Orthopaedic Surgeons on OrthoInfo and additional studies available on PubMed.

### 3.3. DISCERN Score

The mean DISCERN score for answers given by ChatGPT version 3.5 was 44.59 (range 37–51), out of a maximum score of 80, with an ICC of 0.67. The mean DISCERN score for answers given by ChatGPT version 4 was 48.74 (range 36–62), out of a maximum score of 80, with an ICC of 0.83. ChatGPT version 4 had a significantly higher DISCERN score than version 3.5 (*p* < 0.0001). Additionally, a significant between-group difference was found concerning the mean score per DISCERN category, in favor of ChatGPT version 4 (ChatGPT version 4: 3.046 [95% C.I. 2.971–3.121] vs. ChatGPT version 3.5: 2.898 [95% C.I. 2.812–2.984], *p* < 0.0001).

Regardless of the platform used, both ChatGPT versions 3.5 and 4 scored highly in categories relating to response relevance, producing informative and unbiased responses, as well as consistently highlighting the importance of shared decision-making.

ChatGPT version 4 was found to be superior to ChatGPT version 3.5 in relation to the frequency of source citation, as well as the improved provision of external sources of support and information for patients. The mean DISCERN score for questions relating to source citation for ChatGPT version 3.5 was 1.3 compared with a mean score of 2.3 for ChatGPT version 4 (*p* < 0.001).

Overall, 13 (56%) responses given by ChatGPT version 4 provided resources available for patient review, with some sources cited in multiple responses. Of the resources cited, 7 (53%) were direct links to PubMed^®^ indexed studies, comprising of one randomized control trial (RCT), one retrospective study, three literature reviews, and two meta-analyses [23,24,25,26,27,28,29]. The remaining sources cited by ChatGPT consisted of a link to patient education material generated by the American Academy of Orthopaedic Surgeons (AAOS), one private website dedicated to patient education with physician-written, peer-reviewed articles, as well as four websites by privately run US-based orthopedic practices [30,31,32,33,34,35].

### 3.4. Readability

**Figure 1:**
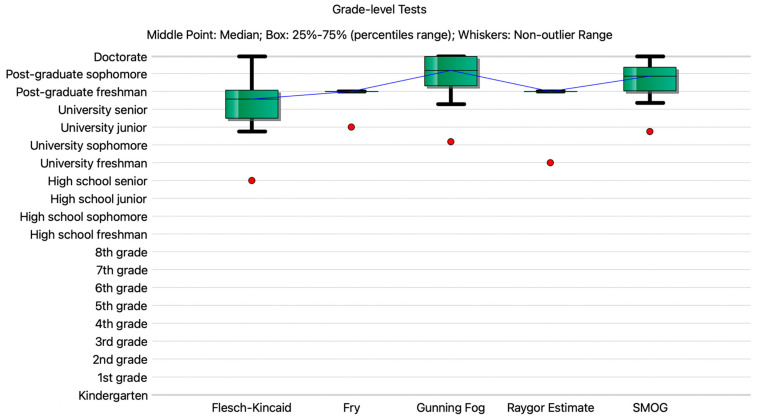
Reading grade level for ChatGPT version 3.5. The horizontal line denotes the median; the upper and lower bounds of each box depict the interquartile range; the whiskers show the lower and upper quartiles; the circles indicate outliers.

**Figure 2:**
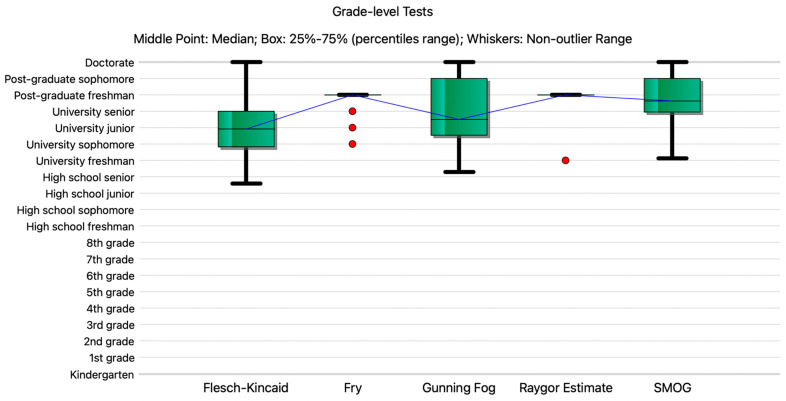
Reading grade level for ChatGPT version 4. The horizontal line denotes the median; the upper and lower bounds of each box depict the interquartile range; the whiskers show the lower and upper quartiles; the circles indicate outliers.

The mean RGL of the questions posed by the investigators was 6.1, below the average 8^th^-grade reading level of the general American public and in keeping with the recommended RGL for patient education materials (PEMs). The mean RGL for answers given by ChatGPT version 3.5 was 17.18 (range, 12–19) (Figure 1 and Table 1), while the mean RGL of ChatGPT version 4 was 16.36 (range, 11.6–19) (Figure 2 and Table 2).

No significant difference was observed between the mean RGL of ChatGPT versions 3.5 and 4 (*p* = 0.136). Of the answers given by both ChatGPT version 3.5 and ChatGPT version 4, none (0%) were written at, or below, the recommended 8th grade reading level regardless of the readability test employed. The mean RGL of the answers given by ChatGPT version 3.5 and ChatGPT version 4 exceeded the 8th grade level by an average of 9.18 grade levels and 8.36 grade levels, respectively (*p* < 0.001). The Flesch Reading Ease Score (FRES) of ChatGPT version 3.5 was 19, while ChatGPT version 4 scored 27, both of which were classified as providing “very difficult” documents, which were more suitable for academics or professionals or intended for an audience with a high level of literacy and familiarity with the subject matter (Table 3).

## 4. Discussion

Our research aimed to assess the readability and quality of the information provided by LLMs, like ChatGPT, concerning the role of platelet-rich plasma (PRP) in the management of OA. ChatGPT has been previously shown to produce good quality information in relation to shoulder stabilization surgery, as well as anterior cruciate ligament injury and treatment [13,15]. Furthermore, ChatGPT has shown promise in the field of hip and knee arthroplasty. ChatGPT responses have been found to demonstrate a high concordance rate with the American Academy of Orthopedic Surgeons’ guidelines concerning the management of hip and knee OA [36]. Kienzle et al. showed that ChatGPT could provide a valuable supplement to informed consent for patients prior to total knee arthroplasty [14]. However, the application of LLMs, like ChatGPT, in regenerative medicine presents a unique challenge due to the prevalence of misinformation and unverified claims online, particularly concerning the indications for, and potential benefits of, PRP therapy. Online resources about PRP have been found to often lack accuracy and are frequently presented at a reading level beyond that of the general population, contributing to confusion and misinformation [5]. Furthermore, the inherent risk of bias in the content generated by LLMs is significant, stemming from their reliance on “unsupervised training” models. This training model exposes LLMs to a wide array of data, mixing high-quality, evidence-based medical information with unverified and potentially misleading claims. Consequently, the information provided by LLMs on contentious subjects, like PRP therapy, demands careful scrutiny and validation.

The DISCERN criteria were used to evaluate the quality of the information provided by both ChatGPT versions 3.5 and 4. We found that both ChatGPT versions 3.5 and 4 delivered information of moderate quality regarding the role of PRP therapy in the management of OA. Both models scored consistently well with respect to response relevance, as well as the provision of balanced and unbiased responses. Furthermore, both models placed particular emphasis on the importance of shared decision making and consultation with a healthcare provider. The answers provided by ChatGPT version 4 were significantly better than ChatGPT version 3.5 (Table 1). This is not surprising as the maturation of the ChatGPT model has led to an estimated 60% improvement in the response quality across models [37]. ChatGPT version 4 significantly outperformed its predecessor with respect to source citation. Overall, 56% of responses given by ChatGPT version 4 contained links to the sources used for the response generation. Of the sources listed, over half (53%) were links to peer-reviewed scientific research, available on PubMed^®^. The enhancement in the precision of source citation from ChatGPT version 3.5 to ChatGPT version 4 marks a significant advancement in the potential for widespread use of LLMs for patient education. Clear and accurate source citation holds crucial importance in patient education, as it allows both patients and physicians to independently verify the scientific validity of the information presented. Healthcare providers and patient organizations should work closely with developers to ensure that future iterations of ChatGPT, or other LLMs developed for patient education, place significant emphasis on the use of pre-screened high-quality sources, which are accessible by the end user. Responses that are well cited not only boost the credibility and reliability of the information, but also empower physicians to recommend these resources with greater certainty.

The second key aim of our research was to assess the readability of the information generated by ChatGPT. Our analysis revealed that ChatGPT version 3.5 had an average reading grade level (RGL) of 17.18, while ChatGPT version 4’s average was slightly lower, at 16.36. Both models significantly surpassed the 8th grade reading level recommended for patient education materials (PEMs), with the level being exceeded by 9.18 and 8.36 grade levels for ChatGPT versions 3.5 and 4, respectively. Prior research has found that the average Flesch–Kincaid Score for internet-based resources relating to PRP therapy is 12.6, lower than the information produced by the ChatGPT models; however, still exceeding the literacy level of the general public [5]. The production of information that significantly exceeds the advised RGL, creates a significant barrier to resource utilization, which makes the widespread utility of LLMs in patient education unlikely. Online resources pertaining to the use of PRP therapy are more accessible to the general public; however, as previously discussed, they are typically of poor quality and are often misleading. Future iterations of ChatGPT must aim to optimize accuracy and readability to optimize their utility for the general public.

## 5. Conclusions

Our study examined the readability and quality of the information provided by language models, specifically ChatGPT versions 3.5 and 4, concerning platelet-rich plasma (PRP) therapy for knee osteoarthritis. We found that both versions deliver information of moderate quality. Notably, ChatGPT version 4 has improved in regard to sourcing accuracy, predominantly citing peer-reviewed, PubMed^®^-indexed scientific research, which demonstrates significant advancements in using LLMs for patient education and health literacy enhancement.

A significant challenge identified through our analysis is the complexity of the text generated by these models, which consistently and significantly surpasses the recommended 6th grade reading level for patient education materials (PEMs). This complexity can restrict the comprehensibility and accessibility of information for the general public, potentially limiting the effectiveness of such tools in patient education.

To address these findings, we suggest several strategies to refine the utility of LLMs, like ChatGPT, for use in medical communication. Future iterations of ChatGPT should aim to simplify the language used to ensure the content is accessible at, or below, the 6th grade reading level, enhancing the readability, without compromising the depth and accuracy of the information. Collaborations between developers, healthcare providers, and patient organizations are essential to regularly update and review the content, ensuring it remains current and scientifically accurate. Moreover, efforts should continue to improve the transparency with which sources are cited, making it easier for patients and clinicians alike to verify the scientific validity of the information provided.

Through these comprehensive measures, LLMs like ChatGPT can evolve into more reliable and practical tools in patient education, empowering users with information that supports informed healthcare decisions, while improving digital health literacy.

## Figures and Tables

**Table 1:** Readability assessment of ChatGPT version 3.5.

Test	Minimum	Maximum	Range	Mean	Std Dev
Flesch–Kincaid	12	19	7	16.3	1.33
Fry	15	17	2	17	0.41
Gunning Fog	14.2	19	4.8	17.8	1.07
Raygor Estimate	13	17	4	17	0.83
SMOG	14.8	19	4.2	17.8	1.00

**Table 2:** Readability assessment of ChatGPT version 4.

Test	Minimum	Maximum	Range	Mean	Std Dev
Flesch–Kincaid	11.6	19	7.4	15	1.88
Fry	14	17	3	17	0.75
Gunning Fog	12.3	19	6.7	16	2.12
Raygor Estimate	13	17	4	17	0.83
SMOG	13.1	19	5.9	16.8	1.38

**Table 3:** Flesch Reading Ease Score for ChatGPT version 3.5 and 4.

Model	Minimum	Maximum	Mean	Std Dev
ChatGPT version 3.5	3	42	19	7.93
ChatGPT version 4	8	40	27	8.30

## Data Availability

The data are available from the corresponding author upon reasonable request.

## Abbreviations

OA—osteoarthritis, PRP—platelet-rich plasma, ACL—anterior cruciate ligament, AI—artificial intelligence, PEMs—patient education materials, FRES—Flesch Reading Ease Score, LLMs—large language models, US—United States, FKGL—Flesch–Kincaid Reading Grade Level, RGL—reading grade level, ICC—intraclass correlation coefficient, AAOS—American Academy of Orthopaedic Surgeons, SMOG—Simple Measure of Gobbledygook.

## Appendix A. Questions

(1) How does PRP work for my knee arthritis?
(2) Does PRP improve knee pain in arthritis?
(3) Will PRP reduce the knee stiffness for my knee arthritis?
(4) Will PRP improve the function with my knee arthritis?
(5) Are PRP injections better than cortisone injections for my knee arthritis?
(6) Are PRP injections better than hyaluronic acid injections for my arthritis?
(7) Will PRP injections help me avoid a knee replacement in the future?
(8) Will PRP injections slow down or limit the progression of arthritis?
(9) Does PRP cause my cartilage to re-grow?
(10) Does PRP stop my cartilage loss?
(11) For how long does PRP work for my knee arthritis?
(12) How many PRP injections will I need for my knee arthritis?
(13) Does PRP work best if I have mild, moderate, or severe arthritis?
(14) Should I get PRP therapy if I have advanced knee arthritis?
(15) Is there evidence for the long-term benefits of PRP therapy for my knee arthritis?
(16) When should someone with arthritis get PRP?
(17) When should someone with arthritis definitely not get PRP injections?
(18) What are the indications for PRP injections in arthritis?
(19) What other treatments can I get for knee arthritis instead of PRP?
(20) Can my knee arthritis be treated with PRP?
(21) What are the potential benefits of PRP in knee arthritis?
(22) What are the potential risks of PRP in knee arthritis?
(23) What does my surgeon hope to achieve by using PRP on my knee arthritis?

## Appendix B

**Table A1:** Summary of Readability Formulae.

Readability Test	Score Type	Description	Formula
Flesch–Kincaid Reading Grade Level	Grade Level	Designed for technical documents as part of the Kincaid Navy personnel collection of tests. Applicable to broad array of disciplines.	G = (11.8 × (B/W)) + (0.39 × (W/S)) – 15.59
Flesch–Kincaid Reading Ease	Index Score (0–100)	The standard test used by many US government agencies. Originally designed to assess newspaper readability. Best suited to school textbooks and technical documents. Scored from 0 to 100, with higher scores indicating easier readability.	I = (206.835 − (84.6 × (B/W)) − (1.015 × (W/S)))
The Raygor Estimate	Grade Level	Designed for most text, including literature and technical documents.	Calculated using the mean number of sentences and long words (≥6 characters) per 100 words, which are plotted on to an RE graph, where the intersection determines the RGL.
Fry	Grade Level	Designed for most text, including technical documents and literature, across a range of levels, from primary school to university level. Best suited to educational text.	Calculated using the mean number of sentences and syllables per 100 words, which are plotted on to a Fry graph, where the intersection determines the RGL.
SMOG	Grade Level	Assesses for 100% comprehension, whereas most formulae test for roughly 50–75% comprehension. Applicable to secondary age (4th grade to college) level text. Most accurate when applied to documents ≥ 30 sentences in length.	G = 1.0430 × √C + 3.1291
FORCAST	Grade Level	The only test not designed for running narrative. Developed to assess US army technical manuals and forms.	G = 20 − (M/10)
Gunning Fog	Grade Level	Applicable to numerous disciplines. Originally designed for American businesses to improve the readability of their writing.	G = 0.4 × (W/S + ((C*/W) × 100))

G = grade level; B = number of syllables; W = number of words; S = number of sentences; RGL = reading grade level; I = Flesch Index Score; RE = Raygor Estimate; SMOG = Simple Measure of Gobbledygook; C = complex words (≥3 syllables); E = predicted Cloze percentage = 141.8401 − (0.214590 × number of characters) + (1.079812 × S); M = number of monosyllabic words; C* = complex words with exceptions including proper nouns, words made of three syllables by the addition of “ed” or “es”, compound words made of simpler words.
